# Combating Acute Myeloid Leukemia via Sphingosine Kinase 1 Inhibitor-Nanomedicine Combination Therapy with Cytarabine or Venetoclax

**DOI:** 10.3390/pharmaceutics16020209

**Published:** 2024-01-31

**Authors:** Thao M. Nguyen, Paul Joyce, David M. Ross, Kristen Bremmell, Manasi Jambhrunkar, Sook S. Wong, Clive A. Prestidge

**Affiliations:** 1 Centre for Pharmaceutical Innovation, UniSA: Clinical and Health Sciences, University of South Australia, Adelaide, SA 5001, Australia; thao.nguyen2@unisa.edu.au (T.M.N.); paul.joyce@unisa.edu.au (P.J.); kristen.bremmell@unisa.edu.au (K.B.); mantri.manasi6@gmail.com (M.J.); sooksan@live.com (S.S.W.); 2 Adelaide Medical School, University of Adelaide, Adelaide, SA 5001, Australia; david.ross@sa.gov.au; 3 Department of Haematology, Flinders University and Medical Centre, Adelaide, SA 5001, Australia; 4 Department of Haematology and Bone Marrow Transplantation, Royal Adelaide Hospital, Adelaide, SA 5001, Australia; 5 Centre for Cancer Biology, University of South Australia and SA Pathology, Adelaide, SA 5001, Australia

**Keywords:** anti-cancer activity, targeted cancer therapy, combination therapy, drug resistance, acute myeloid leukemia, sphingosine kinase 1 inhibitors, liposomal drug encapsulation, cytarabine, venetoclax, nanocarrier drug delivery

## Abstract

MP-A08 is a novel sphingosine kinase 1 (SPHK1) inhibitor with activity against acute myeloid leukemia (AML). A rationally designed liposome-based encapsulation and delivery system has been shown to overcome the physicochemical challenges of MP-A08 and enable its effective delivery for improved efficacy and survival of mice engrafted with human AML in preclinical models. To establish therapies that overcome AML’s heterogeneous nature, here we explored the combination of MP-A08-loaded liposomes with both the standard chemotherapy, cytarabine, and the targeted therapy, venetoclax, against human AML cell lines. Cytarabine (over the dose range of 0.1–0.5 µM) in combination with MP-A08 liposomes showed significant synergistic effects (as confirmed by the Chou–Talalay Combination Index) against the chemosensitised human AML cell lines MV4-11 and OCI-AML3. Venetoclax (over the dose range of 0.5–250 nM) in combination with MP-A08 liposomes showed significant synergistic effects against the chemosensitised human AML cell lines, particularly in venetoclax-resistant human AML cells. This strong synergistic effect is due to multiple mechanisms of action, i.e., inhibiting MCL-1 through SPHK1 inhibition, leading to ceramide accumulation, activation of protein kinase R, ATF4 upregulation, and NOXA activation, ultimately resulting in MCL-1 degradation. These combination therapies warrant further consideration and investigation in the search for a more comprehensive treatment strategy for AML.

## 1. Introduction

Acute myeloid leukemia (AML) is an aggressive blood cancer originating from the uncontrolled growth of immature myeloid cells in the red bone marrow and peripheral blood [1], leading to dysfunctional hematopoiesis [1,2]. Diagnosis involves assessing cell morphology, immunophenotype, cytogenetics, and the mutational profile [3]. Despite the tremendous heterogeneity of AML characterised by many sub-types and mutations [4], most patients receive one of two standard chemotherapy approaches, and, if needed, allogeneic hematopoietic stem cell transplantation for patients who have displayed a high risk of relapse [4]. Since the 1970s, the above-mentioned standard care for younger patients has not evolved, with induction using cytarabine and an anthracycline, followed by consolidation chemotherapy with higher doses of cytarabine [2,5]. Despite its efficacy in achieving remission, the current approach often results in short-lived responses, leading to an unfavourable overall prognosis with a five-year survival rate of around 40–45% in adults below sixty and less than 10% in elderly patients. The addition of the BCL-2 inhibitor, venetoclax, to either zacytidine or low-dose cytarabine has emerged as a standard treatment for older patients [6], but the improvement in median survival with this approach was <6 months [7]. The persistence of leukemic stem/progenitor cells (LSPCs) that are resistant to therapy leads to relapse, necessitating the development of innovative approaches for AML treatment to improve longer-term outcomes.

Sphingosine kinase 1 (SPHK1) is an enzyme known for its pro-survival signalling roles in cancer initiation, progression, and resistance to cytarabine chemotherapy [8,9]. Sphingolipids play a crucial role in determining cell fate [10,11], with sphingosine and ceramide acting as pro-apoptotic molecules, while sphingosine-1-phosphate (S1P) enhances cell proliferation and survival [12]. The dynamic balance between ceramide and SP1, referred to as the “sphingolipid rheostat”, is regulated by SPHK1 [8,12,13]. Elevated SPHK1 levels lead to dysregulation of this balance, promoting cell survival, angiogenesis, and oncogenesis [14,15]. Studies have demonstrated that SPHK1 is over-expressed and activated in AML cells. Targeting SPHK1 with the selective inhibitor MP-A08 induces caspase-dependent apoptotic cell death in AML cell lines, primary AML patient cells and LSPCs, while sparing normal hematopoietic progenitor cells [8,16,17]. In mouse models, MP-A08 reduces the leukemic burden and improves survival in AML xenografts [16,17,18]. The findings suggest that MP-A08, as a selective SPHK1 inhibitor, holds promise as a potential anti-cancer therapy for AML. Most significantly, MP-A08 demonstrates enhanced killing of primary AML blast cells when combined with the first-line induction chemotherapy cytarabine [18]. This indicates its potential not only as a monotherapy but also in combination with standard cytarabine chemotherapy as an effective AML treatment.

In addition to conventional cytotoxic chemotherapies, more targeted therapies have been developed to treat AML. Venetoclax, approved by the FDA in 2017, is a selective BCL-2 inhibitor that triggers the intrinsic apoptosis pathway [19]. AML cells often express high levels of the B-Cell Lymphoma 2 (BCL-2) pro-survival protein that promotes leukemogenesis [19,20,21]. However, venetoclax monotherapy is less effective in AML [22,23], as many AML cells also express high levels of another pro-survival protein, induced myeloid leukemia cell differentiation protein (MCL-1), which venetoclax does not target [24,25,26]. MCL-1 is commonly upregulated in AML cells and confers resistance to venetoclax [19]. Therefore, novel strategies to target both BCL-2 and MCL-1 in combination have excellent potential to improve response in AML [19,20].

The anti-cancer agent MP-A08, as a free compound, has shown promise by significantly inhibiting MCL-1 expression in AML. MP-A08 induces AML cell apoptosis by inhibiting MCL-1 through SPHK1 inhibition, leading to ceramide accumulation, the activation of protein kinase R, the upregulation of activating transcription factor 4 (ATF4), and the activation of phorbol-12-myristate-13-acetate-induced protein 1 (Noxa), ultimately resulting in MCL-1 degradation [16]. These findings suggest that combining MP-A08 and venetoclax may improve AML patient response rates, presenting an innovative, combinatorial therapeutic approach for AML treatment.

Despite the potential for MP-A08 to serve as an anti-AML therapy, either as a monotherapy or in combination, the low solubility and limited in vivo bioavailability of MP-A08 hinder its efficacy and potential for clinical implementation. To overcome this challenge, recent work in our laboratory demonstrated that a novel approach of encapsulation and delivery of MP-A08 in liposomal formulations exhibited enhanced potency and efficacy against AML cells in vitro and in vivo [17]. The utilisation of nanocarriers to encapsulate anti-cancer agents has been shown to improve drug potency and efficacy in targeting cancer cells [22,23,24,25,27]. Liposomes are ‘gold standard’ nanoparticles used as drug-delivery systems due to their desirable characteristics and promising preclinical and clinical results [26,28,29]. Liposomes can encapsulate hydrophobic and/or hydrophilic therapeutic agents, resulting in improved drug solubility and pharmacokinetic and pharmacodynamic profiles [5,30,31]. The efficacy of the MP-A08-loaded liposome-based drug-delivery system that we have recently developed could be attributed to improvements in solubility, bioavailability, and bone marrow biodistribution of MP-A08, which lead to significantly prolonged survival of mice engrafted with human AML cancer [17]. Building on these recent advances, in this study, we investigate the efficacy of MP-A08-loaded liposomes in combination with cytarabine or venetoclax through in vitro studies using the human AML cell line MV4-11 and the venetoclax-resistant human AML cell line OCI-AML3.

## 2. Materials and Methods

AML Cell Lines: the human AML cell lines used in this study were OCI-AML3, obtained from the Leibniz Institute DSMZ in Braunschweig, Germany, and MV4-11 cells, acquired from the American Type Culture Collection (ATCC) in Manassas, VA, USA. Culturing of the cell lines took place in Roswell Park Memorial Institute (RPMI) 1640 Medium, obtained from Gibco—ThermoFisher Scientific in Waltham, MA, USA. The medium was supplemented with 10% Fetal Bovine Serum (FBS) from HyClone Cytiva Life Sciences in MA, USA, and 1% Penicillin and Streptomycin (P/S) from Gibco—ThermoFisher Scientific. The cells were incubated under a humidified atmosphere of 5% CO_2_ at 37 °C in a Panasonic Healthcare Co. incubator from Osaka, Japan. To maintain sterility, all cell culture procedures were executed within a Class II Biohazard Cabinet (Class II, Safety Cabinet, ThermoFisher Scientific, Dreieich, Germany). Centrifugation procedures were carried out using an Eppendorf 5810R centrifuge from Germany. For precise cell seeding density, cell counting was performed with 0.4% trypan blue staining obtained from Sigma Aldrich in St. Louis, MO, USA. Sub-culturing of cells occurred every 2 to 3 days, and the cell lines were maintained within specific ranges: 1 × 10^5^–1 × 10^6^ cells/mL for MV4-11 and 0.5–2.0 × 10^6^ cells/mL for OCI-AML3.

Research compounds and drugs: MP-A08 (CAS 219832-49-2) was synthesised, purified, and identity-verified using ChemBridge Inc. (San Diego, CA, USA). Cytarabine (CAS 147-94-4) was purchased from Calbiochem (San Diego, CA, USA) and venetoclax (CAS 1257044-40-8) from Active Biochem (Kowloon, Hong Kong).

Preparation of the MP-A08 Liposomal Formulation: The MP-A08 liposomal formulation was synthesised as previously described [17]. In brief, liposomes were prepared using 1,2-dimyristoyl-sn-glycero-3-phosphocholine (DMPC) and 1,2-dipalmitoyl-sn-glycero-3-phospho-(1′-rac-glycerol) (sodium salt, DPPG), both purchased from Avanti Polar Lipids (Alabaster, AL, USA). Propanol and methanol were obtained from Merck (Bayswater, VIC, Australia). Liposomal formulations were created using the NanoAssemblr^®^ benchtop system (ATA Scientific, New South Wales, Australia). To prepare the liposomes, DMPC (390 mg), DPPG (1.5 mg), and MP-A08 were dissolved in propanol and methanol (9:1, 10 mL). The organic phase (containing phospholipids and drug) and the aqueous phase (phosphate-buffered saline (PBS)) flowed through each channel of the NanoAssemblr^®^ microfluidic cartridge at 12 mL/min, with a lipid-to-PBS mixing ratio of 1:2. Unloaded MP-A08 was separated from MP-A08-loaded liposomes using a 10,000 kDa ultrafiltration unit at 3000 rpm for 30 min. To concentrate the liposomal formulation, MP-A08-loaded liposomes were centrifuged using high-speed centrifugation at 20,000 rpm for 15 min, and the resulting pellet was resuspended with PBS. The quantitation of MP-A08-loaded within the liposomes was analysed by liquid-chromatography mass spectrometry (LC/MS—Agilent 6550 iFunnel Q-TOF LC/MS—San Francisco, CA, USA), as previously described [17]. The mean particle size, zeta potential, and polydispersity index (PDI) of the liposomes were measured using dynamic light scattering (DLS) with the Malvern Zetasizer Nano ZS (Worcestershire, UK).

Cell Viability Assay: Cells were seeded in 48-well plates at a density of 1 × 10^5^ cells per well, supplemented with 0.5% FBS and 1% P/S in RPMI 1640. They were then treated with various doses of MP-A08-loaded liposomes, venetoclax, cytarabine, or combination therapies. Doses of venetoclax, cytarabine, and MP-A08-loaded liposomes were chosen according to previous studies [16,17,18]. For combination therapies, cells were pre-treated with venetoclax or cytarabine for 30 min before the addition of MP-A08-loaded liposomes. Cell survival was determined after 48 h by annexin V fluorescein isothiocyanate (FITC; BD Biosciences) negativity and propidium iodide (PI; BD Biosciences) exclusion using the LSR Fortessa flow cytometer. Cell viability was analysed using the BD LSRFortessa™ Cell Analyzer (BD BioSciences, San Jose, CA, USA), and the percentage of cell viability was assessed using FCS Express 6 Flow Research Edition software, version 6.

Statistical Analysis: We conducted statistical analyses using GraphPad Prism 8 software (San Diego, CA, USA) to determine the half-maximal inhibitory concentration (IC50) of the drug. Treated groups were compared with their corresponding controls using a two-tailed unpaired Student’s *t*-test. A significance level of less than 0.05 (*p* < 0.05) was considered statistically significant. The evaluation of drug synergy for combination therapies followed a previously described method [16,18], employing the Chou–Talalay Combination Index (CI) method. This analysis was performed using CompuSyn software, version 6 (ComboSyn Inc, Paramus, NJ, USA) based on the median-effect principle of the mass-action law. The drug effects were quantified as synergism (CI < 1), additive effect (CI = 1), and antagonism (CI > 1).

## 3. Results

### 3.1. MP-A08-Loaded Liposomes Combined with Cytarabine Provide a Synergistic Effect against AML Cells

**Figure 1:**
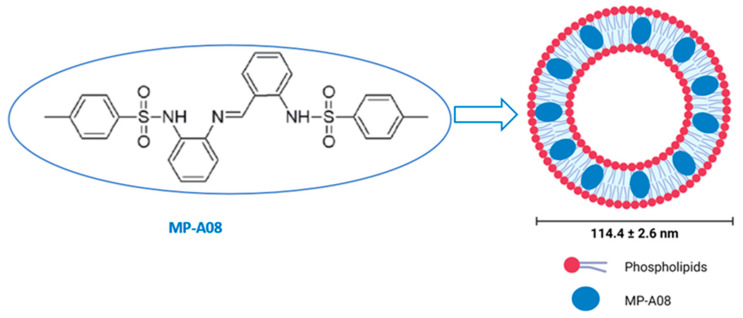
Encapsulation of MP-A08 free compound in liposomes. MP-A08 structure showing hydrophobicity. Liposomes embedding MP-A08 in the hydrophobic region.

The hydrophobic nature of the anti-cancer MP-A08 agent hinders its in vivo delivery and, subsequently, its clinical translation (Figure 1). For this reason, the encapsulation of the MP-A08 agent in the nanocarrier, MP-A08 liposomes, was designed and prepared using microfluidics (the NanoAssemblr^®^ benchtop system) as previously described [17]. Briefly, the engineered phospholipid bilayer of MP-A08 liposomes comprised a mixture of amphipathic and cationic lipids. The mean diameter of the liposomes was 114 ± 2.6 nm with a net negative surface potential of −10 mV.

Cytarabine, a pyrimidine nucleoside analogue, is a standard first-line chemotherapeutic used in induction chemotherapy for AML [4,32]. Previous studies showed that MP-A08, as a free compound, chemosensitises AML cells to cytarabine [18]. We recently demonstrated that MP-A08 encapsulated in liposomes exhibits significantly enhanced potency and efficacy against AML cells in vitro and prolongs the survival of mice harbouring human AML cancer [17]. In this study, we conducted experiments to investigate the synergistic effect of MP-A08-loaded liposomes in combination with cytarabine.

**Figure 2:**
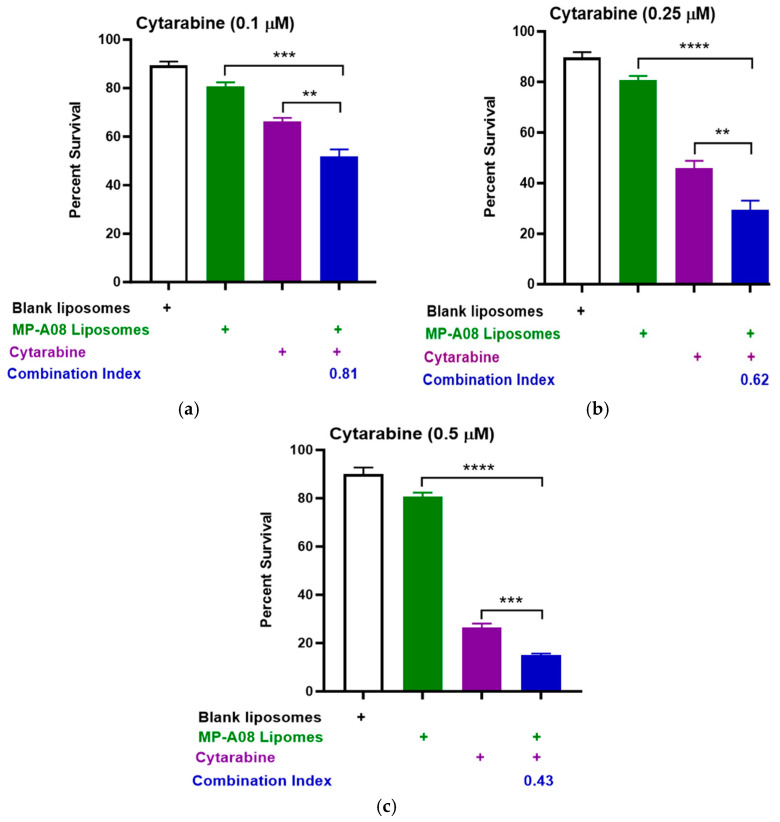
MP-A08-loaded liposomes chemosensitised human AML cell line MV4-11 to cytarabine. Cell viability assays of cytarabine in combination with MP-A08-loaded liposomes were assessed in MV4-11 cell line. MV4-11 was treated with blank liposomes, MP-A08-loaded liposomes (0.125 µM), and cytarabine [(**a**) 0.1 µM, (**b**) 0.25 µM, (**c**) 0.5 µM] as a single agent or in combination with MP-A08-loaded liposomes for 48 h. Cell viability was assessed using annexin V and PI staining by flow cytometry. Each experiment contained three replicates for every drug combination. Error bars indicate the mean ± range of 3 replicates of representative experiment of *n* = 3 experiments. Synergism was determined by the Chou–Talalay Combination Index (CI) using CompuSyn software. CI values are shown for distinct dose combinations of MP-A08-loaded liposomes and cytarabine. *p* values were calculated using a two-tailed Student’s *t*-test, ** *p* < 0.01, *** *p* < 0.001 and **** *p* < 0.0001.

Titration of cytarabine in combination with MP-A08-loaded liposomes was performed. The data showed that MP-A08-loaded liposomes in combination with cytarabine resulted in synergistic apoptotic cell death in the human AML cell line MV4-11 in vitro (Figure 2). AML is a typically lethal molecularly heterogeneous disease, where MV4-11 is the most used AML cell line to determine drug efficacy, due to the cells bearing most common AML mutations such as FLT3-ITD, TP53, and MLL-AF4 fusion oncogene. The drug synergism is assessed by Combination Index (CI) values where CI values less than 1 indicate synergism, CI values equal to 1 indicate additive effects, and CI values greater than 1 indicate antagonism [33]. Importantly, the lower the CI values from 1, the greater the synergistic effect. We observed that blank liposomes (i.e., liposomes without MP-A08 being loaded) exhibited no toxicity toward human AML cells, which aligned with a previous study [17]. Next, the data showed that MP-A08 liposomes alone demonstrated a minor reduction in cell viability, a result consistent with our previously published work [17]. The impact of cytarabine on human AML cells became more pronounced as the titration concentration increased from 0.1 µM (Figure 2a) to 0.25 µM (Figure 2b) and 0.5 µM (Figure 2c), resulting in increased apoptotic cell death of approximately 50%, 68%, and 82%, respectively. These findings align with findings from a prior published study [18]. A notable discovery here is that, in the presence of MP-A08 liposomes, the efficacy of cytarabine is significantly enhanced, indicating a synergistic effect, as evidenced by a CI index of less than 1.

**Figure 3:**
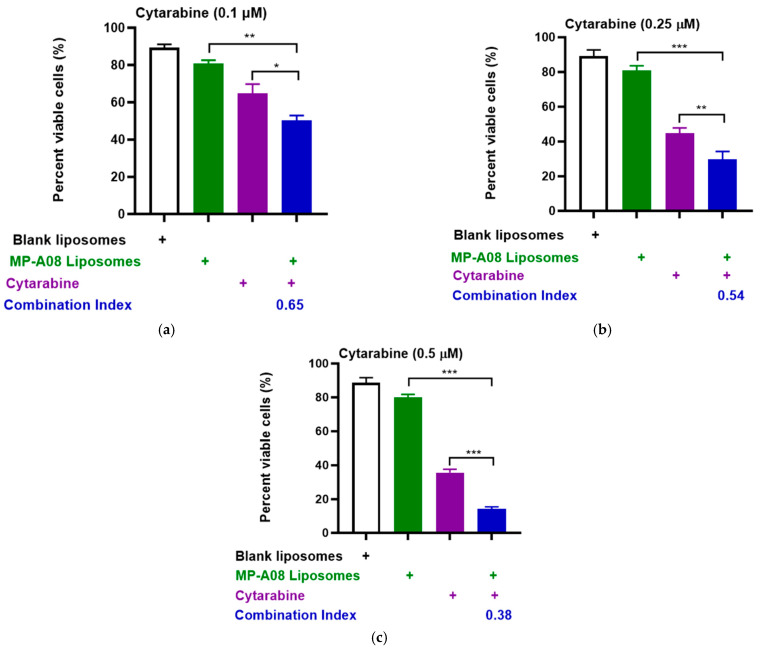
MP-A08-loaded liposomes chemosensitised human AML cell line OCI-AML3 to cytarabine. Cell viability assays of cytarabine in combination with MP-A08-loaded liposomes were assessed in OCI-AML3 cell line. OCI-AML3 was treated with blank liposomes, MP-A08-loaded liposomes (0.125 µM), and cytarabine [(**a**) 0.1 µM, (**b**) 0.25 µM, (**c**) 0.5 µM] as a single agent or in combination with MP-A08-loaded liposomes for 48 h. Cell viability was assessed using annexin V and PI staining by flow cytometry. Each experiment contained three replicates for every drug combination. Error bars indicate the mean ± range of 3 replicates of representative experiment of *n* = 3 experiments. Synergism was determined by the Chou–Talalay Combination Index (CI) using CompuSyn software. CI values are shown for distinct dose combinations of MP-A08-loaded liposomes and cytarabine. *p* values were calculated using a two-tailed Student’s *t*-test, * *p* < 0.05, ** *p* < 0.01 and *** *p* < 0.001.

To extend these results obtained in the MV4-11 human AML cell line, we performed more rigorous analyses by testing synergy in another human AML common cell line, OCI-AML3, which carries additional AML mutations such as DNA methyltransferase 3 alpha (DNMT3A), Nucleophosmin 1 (NPM1), and neuroblastoma ras viral oncogene homolog (NRAS). Consistent with previous findings, the drug combination demonstrated apparent synergy (Figure 3). The synergistic enhancement of cell death in AML cells with cytarabine at a concentration of 0.1 µM was approximately 53%, at 0.25 µM, it was around 70%, and at 0.5 µM, it reached approximately 90%. These findings strongly indicate a synergistic increase in apoptotic cell death in AML cells with the combined treatment of cytarabine and MP-A08-loaded liposomes. Collectively, the results suggested that MP-A08-loaded liposomes sensitised human AML cells to cytarabine and enhanced the efficacy of cytarabine in inducing AML cell apoptosis.

### 3.2. MP-A08-Loaded Liposomes Potentiated the Anti-Leukemic Activity of Venetoclax in AML Cells

**Figure 4:**
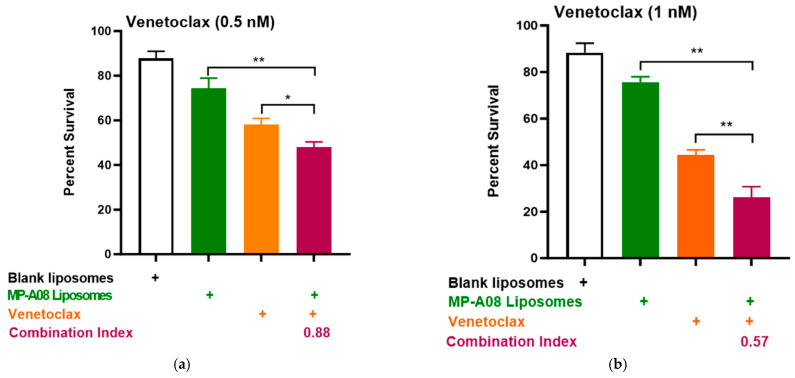

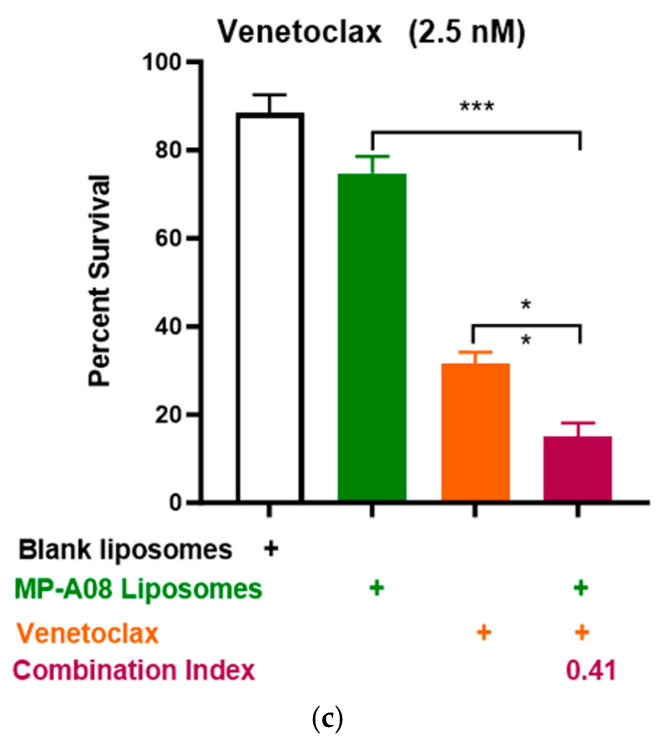
MP-A08-loaded liposomes potentiated venetoclax anti-leukemic activity in MV4-11 AML cell line. Cell viability assays of venetoclax in combination with MP-A08-loaded liposomes were assessed. Representative data of *n* = 3 experiments, each containing three replicates for every drug combination (error bars indicating the mean ± range of 3 replicates). MV4-11 human AML cell lines were treated with blank liposomes, MP-A08 liposomes (0.125 µM), and venetoclax [(**a**) 0.5 nM, (**b**) 1 nM, (**c**) 2.5 nM] as a single agent or in combination for 48 h. Cell viability was assessed using annexin V and PI staining via flow cytometry. Synergism was determined by the Chou–Talalay Combination Index (CI) using CompuSyn software. CI values are shown for distinct dose combinations of MP-A08-loaded liposomes and venetoclax. *p* values were calculated using a two-tailed Student’s *t*-test, * *p* < 0.05, ** *p* < 0.01 and *** *p* < 0.001.

Building on recent studies demonstrating that MP-A08 (as a free anti-cancer compound) enhanced the efficacy of venetoclax in inducing AML cell death [16], here, we assessed if this activity could be further enhanced by MP-A08-loaded liposomes. We selected the venetoclax-sensitive human AML cell line, MV4-11. A titration of venetoclax in combination with MP-A08-loaded liposomes was performed. The synergistic increase in cell death with the treatment of AML cells showed that at a venetoclax concentration of 0.5 nM, it was approximately 50% cell death, at 1 nM, it was around 70%, and at 0.25 µM, it reached approximately 84% (Figure 4). Most importantly, MP-A08 liposomes at a very low concentration (0.125 µM) had a minimal anti-AML effect; however, the combination of MP-A08-loaded liposomes and venetoclax resulted in a significant reduction in cell viability and promoted synergistic apoptotic cell death in venetoclax-sensitive MV4-11 AML cells, even at a venetoclax concentration as low as 0.5 nM. These data show that MP-A08-loaded liposomes potentiate venetoclax activity in a venetoclax-sensitive human AML cell line.

### 3.3. MP-A08-Loaded Liposomes Overcome Venetoclax Resistance in Human AML Cells

**Figure 5:**
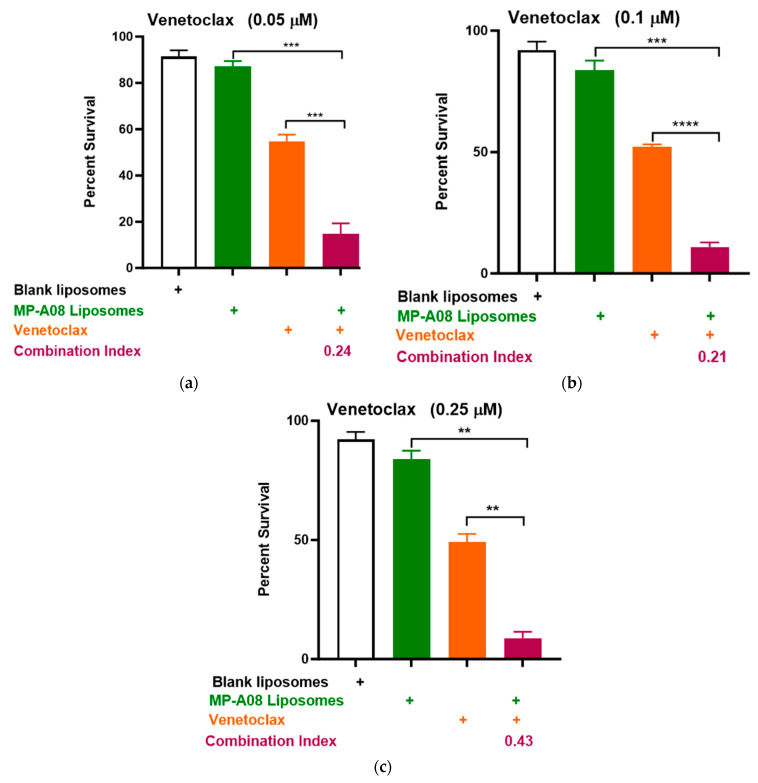
MP-A08-loaded liposomes enhanced venetoclax anti-leukemic activity in venetoclax-resistant AML cell line OCI-AML3. Cell viability assays of venetoclax in combination with MP-A08-loaded liposomes were assessed. Representative data of *n* = 3 experiments, each containing three replicates for every drug combination (error bars indicating the mean ± range of 3 replicates). Venetoclax-resistant human AML OCI-AML3 cells were treated with blank liposomes, MP-A08-loaded liposomes (0.125 µM), or venetoclax [(**a**) 0.05 µM, (**b**) 0.1 µM, (**c**) 0.25 µM] as a single agent or in combination for 48 h. Cell viability was assessed using annexin V and PI staining by flow cytometry. Synergism was determined by the Chou–Talalay Combination Index (CI) using CompuSyn software. CI values are shown for distinct dose combinations of MP-A08-loaded liposomes and venetoclax. *p* values were calculated using a two-tailed Student’s *t*-test, ** *p* < 0.01, *** *p* < 0.001 and **** *p* < 0.001.

In earlier studies, both free and liposomal MP-A08 enhanced the effectiveness of venetoclax in comprehensive in vitro and in vivo studies [17]. To further investigate the potential combinational efficacy of MP-A08-loaded liposomes and venetoclax against venetoclax-resistant human AML cells, a titration of venetoclax was tested in combination with MP-A08-loaded liposomes. The data showed a significant reduction in cell viability and promoted synergistic apoptotic cell death in venetoclax-resistant OCI-AML3 cell lines (Figure 5). The synergistic increase in cell death with the treatment of AML cells indicated that at a venetoclax concentration of 0.05 µM, cell death was approximately 80%, at 0.1 µM, it was around 88%, and at 0.25 µM, it reached approximately 91%. These results demonstrated a significant reduction in cell viability and promoted synergistic cell death in venetoclax-resistant OCI-AML3 cell lines, showing an increase in apoptotic cell death as the concentration of venetoclax increased. For instance, at the highest concentration of venetoclax in combination with MP-A08-loaded liposomes, a 6-fold reduction in cell viability was observed. These results indicated that MP-A08-loaded iposomes significantly abrogated venetoclax resistance in the human AML cell line OCI-AML3.

## 4. Discussion

This study discusses the challenges in treating acute myeloid leukemia (AML) and presents a novel approach using liposomal drug delivery for enhanced efficacy and reduced toxicity. The poor survival rates in AML, especially in older patients, necessitate the development of new therapies. Standard induction chemotherapy with cytarabine often leads to refractory relapse, prompting the search for alternative treatments [34,35,36]. Previous studies demonstrated MP-A08, a sphingosine kinase inhibitor, as a potential anti-AML agent. While previous research demonstrated the synergy of MP-A08 and cytarabine, the hydrophobic nature of MP-A08 hindered clinical translation. Most SPHK1 inhibitors developed to target the sphingosine-binding site of the enzyme consequently suffer from poor specificity and poor “drug-like” properties. The implementation of liposomal-based drug delivery has the potential to significantly improve patient outcomes. Recent evidence of novel liposomal CPX-351 (also known as Vyxeos^®^), a cytarabine and daunorubicin liposomal formulation approved by the FDA to treat newly diagnosed therapy-related AML or AML with myelodysplasia-related changes [23,37,38,39,40,41,42], has emphasized the importance of nanomedicine in the treatment of AML. Indeed, recent work in our laboratory employing a reliable platform of liposomal encapsulation to effectively deliver MP-A08 to eradicate human AML cells engrafted in mice and significantly enhance mice survival was a remarkable breakthrough [17], demonstrating the potential of the MP-A08 liposomal formulation as a promising monotherapy against AML.

Given the heterogeneity and high mortality rates in AML, there is an urgent need for new treatments to improve patient prognosis and overall survival. New therapeutic approaches have been developed to address this challenge, combining existing chemotherapeutics with targeted therapies inhibiting various cell signalling pathways associated with chemoresistance and cancer relapse. This is, in part, due to the cancer stem cell’s ability to differentiate into heterogeneous lineages of cancer cells [2]. Effective combinational therapies include cytarabine combined with sorafenib (FMS-like tyrosine kinase 3—FLT3 inhibitor) [43], ivosidenib and enasidenib (IDH2 inhibitors) [44], glasdegib (a Hedgehog signalling pathway inhibitor) [45], or alvocidib (cyclin-dependent kinase 9 inhibitor) and mitoxantrone [46]. These therapies have shown promising results in addressing AML’s heterogeneity and high mortality rates.

The work presented in this study is the first to successfully demonstrate the combination of MP-A08-loaded liposomes and cytarabine, revealing synergistic apoptotic cell death at significantly lower doses of cytarabine. This approach has the potential to minimize drug toxicity. Notably, previous studies achieved synergistic AML cell death with a combination of cytarabine at 10 µM and free MP-A08 at 10 µM [16]. In contrast, our study utilized MP-A08-loaded liposomes with an MP-A08 concentration of 0.125 µM (80 times less dosing) and cytarabine at 0.1 µM (100 times lower dosing). These findings provide evidence for the enhanced potency and efficacy of both MP-A08 and cytarabine through liposomal delivery compared to MP-A08 as a free compound. Considering the generally poor health condition of leukemia patients, the effective low-dose administration of cytarabine and MP-A08 implies reduced drug toxicity. Standard induction regimes for AML patients often lead to myelotoxicity, resulting in death in at least 25% of cases during chemotherapy induction. Additionally, the complete remission rate is less than 35% in patients over 65 years of age, partly due to an age-related increase in myelotoxicity [47]. Standard-dose cytarabine also presents side effects such as neutropenia, thrombocytopenia, anaemia, fever, skin rash, aches, pains, increased sweating, gastrointestinal toxicity (especially oral mucositis), nausea, vomiting, diarrhea, intestinal ulceration, ileus, and subsequent Gram-negative septicaemia [47]. Moreover, severe and sometimes irreversible cerebellar/cerebral toxicity occurs in 5 to 15% of courses of treatment [47]. The findings in this study suggest that the liposomal drug-delivery platform reduces the effective dosing of both MP-A08 and cytarabine. This offers a promising strategy for future combination therapies employing low-dose cytarabine, potentially producing optimal anti-AML effects with reduced chemotherapy toxicity, thereby potentially saving the lives of patients suffering from cytarabine toxicity.

The last decade has seen significant strides in comprehending AML biology, leading to the emergence of novel targeted therapies that enhance patient outcomes. Notably, alongside the conventional chemotherapy cytarabine, recent years have witnessed rapid shifts in the therapeutic landscape with the FDA’s approval of several new targeted drugs. One such agent is venetoclax, a BH3 mimetic renowned for its efficacy in targeting the anti-apoptotic B-cell leukemia 2 (BCL-2) family proteins [1,48]. BCL2 is an anti-apoptotic protein that enhances leukemic cell survival through the regulation of the mitochondrial apoptotic pathway. An increased level of BCL-2 expression is associated with poor outcomes in patients receiving intensive chemotherapy for AML. For this reason, venetoclax has gained approval as a targeted therapy for AML [6,19,21,49].

However, the challenge of venetoclax resistance looms large, attributed to the minimal potency of venetoclax monotherapy in AML patients resistant to BCL-2 inhibition [50]. Studies underscore the high dependence of AML blast cells and cancer stem cells on MCL-1 for survival and resistance [51,52,53]. MCL-1, an anti-apoptotic BCL2 family member not targeted by venetoclax, is commonly upregulated in AML cells, conferring resistance. The overexpression of MCL-1 sequesters the BH3-only proteins, which prevent BAX/BAK oligomerisation, the release of cytochrome c, and the subsequent activation of the intrinsic apoptosis pathway [54,55]. For instance, the human AML cell line OCI-AML3 acquired resistance to venetoclax due to the overexpression of MCL-1 [54,56]. Therefore, while venetoclax is highly effective in other types of leukemia cancers, in AML, venetoclax encounters MCL-1-dependent resistance. To this end, increasing mechanistic studies in the last few years have indicated that MP-A08 promotes caspase-dependent apoptotic cell death in AML cells, associated with the BCL2 family proteins. Notably, dual targeting of BCL-2 and MCL-1 emerges as a promising strategy to overcome resistance [50,51]. According to Lin et al., targeting MCL-1 potentiated venetoclax activity and augmented the anti-leukemic effects on venetoclax-resistant AML cells [57].

Importantly, studies previously demonstrated that SPHK1 inhibition by MP-A08 significantly induces MCL-1 degradation [16,18], and this highlights a new approach to targeting MCL-1 [18]. Indeed, MP-A08-induced killing of AML cells occurs via MCL-1 inhibition [18], a mechanism whereby SPHK1 inhibition leads to ceramide accumulation, which was found to directly activate protein kinase R, resulting in the activation of ATF4 and upregulation of NOXA, which in turn leads to the degradation of MCL-1 [16]. Thus, targeting MCL-1 through SPHK1 inhibition represents an innovative therapeutic approach in combination with venetoclax for the treatment of AML. Indeed, recent work demonstrated that MP-A08, in combination with venetoclax, exhibits highly synergistic anti-leukemic activity against primary AML patient cells in vitro and significantly prolongs the survival of mice with AML disease [16]. The data suggest that combination therapy of MP-A08 and venetoclax increases the response rates of MCL1-dependent AML.

**Figure 6:**
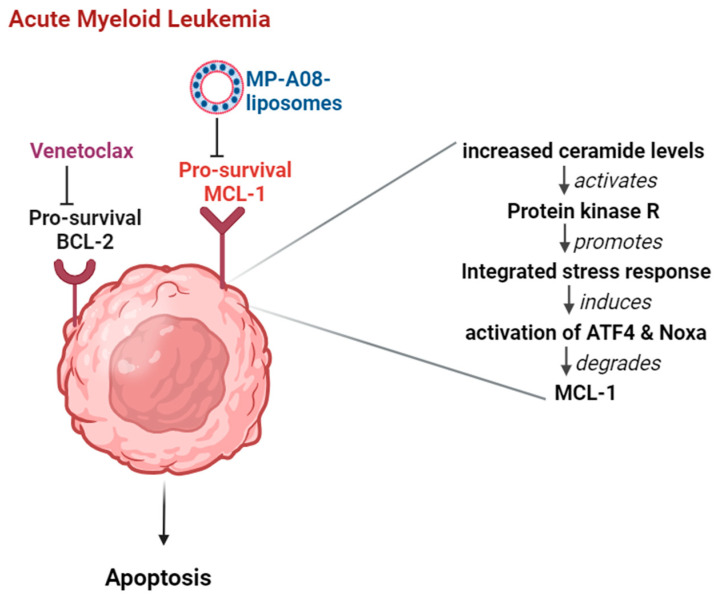
Combination therapy of venetoclax and MP-A08-loaded liposomes synergistically induces apoptotic cell death in AML. The pro-survival proteins BCL-2 and MCL-1 experience upregulation in AML cells. When MP-A08-loaded liposomes bind to MCL-1, ceramide levels in AML cells increase [16]. This elevation activates protein kinase R, subsequently promoting an integrated stress response. This cascade leads to the activation of ATF4 and Noxa, ultimately resulting in the degradation of MCL-1. Once MCL-1 is degraded, venetoclax maximally affects AML cells, inducing apoptosis.

However, as mentioned above, the hydrophobic nature of MP-A08 hinders its clinical translation. Building on our previous breakthrough marking the first introduction of MP-A08 liposomal delivery as a promising monotherapy against AML, this study further demonstrates the potential for combination therapies (Figure 6). The synergistic effect observed between MP-A08 and venetoclax, particularly when delivered through liposomes, showcases enhanced efficacy, particularly with reduced dosage requirements. The observed synergistic outcomes, in agreement with Western blot analyses shown in previous studies [16,18], could be attributed to SPHK1 inhibition and MCL-1 degradation by MP-A08, coupled with BCL-2 inhibition by venetoclax, which, in turn, promote synergistic apoptotic cell death in both venetoclax-sensitive (MV4-11) and -resistant (OCI-AML3) cell lines in vitro. It is crucial to emphasize that AML is typically a lethal and molecularly heterogeneous disease. Most AML cases retain a wild-type TP53 gene, encoding the pro-apoptotic tumour suppressor p53. TP53 mutations are widespread across all domains of the gene, especially within the DNA-binding domain. TP53-mutated AML is associated with a poor response to chemotherapy. Additionally, the MV4-11 human AML cell line contains mutant FLT3-ITD and expresses the MLL-AF4 fusion protein common in AML cancer. The findings in this study align with a previous study that demonstrated apoptosis induction in the most common type of AML cancer using the free MP-A08 compound and venetoclax [16]. Notably, our work reveals that an MP-A08 concentration (loaded in liposomes) of 0.125 µM (i.e., 80 times less dosing) and a venetoclax concentration in combination with MP-A08-loaded liposomes was approximately 4-to-5 times lower, showcasing an impressive outcome.

Venetoclax resistance is an emerging concern in the clinic, prompting the adoption of combination therapies to improve patient survival. To further explore the synergistic effect of venetoclax and MP-A08 liposomes in venetoclax-resistant human AML cell lines, we utilized OCI-AML3 for a more rigorous synergy analysis. OCI-AML3, a venetoclax-resistant human AML cell line, contains NPM1, DNMT3A, and NRAS, and exhibits various TP53 statuses. The drug combination displayed apparent synergy, with an MP-A08 concentration in the liposomal formulation of only 0.125 µM, compared to 10 µM in MP-A08 as a free compound demonstrated in a previous study [16], representing an approximately 80 times lower dosing than MP-A08 used as a free compound. Furthermore, our data demonstrated that MP-A08-loaded liposomes and venetoclax exhibited substantially greater and more consistent synergy in venetoclax-resistant cell lines across a range of drug doses. Collectively, the combination of MP-A08 and venetoclax may offer an effective therapy in patients who are insensitive to venetoclax, reducing the likelihood of treatment failure due to acquired resistance. These findings underscore that liposomal delivery of the anti-cancer compound MP-A08 not only enhances venetoclax efficacy in AML cells but also reduces the required venetoclax dose, potentially minimizing toxicity in patients. Common venetoclax toxicities include myelosuppression, tumour lysis, neutropenia, anaemia, and thrombocytopenia [58,59]. Particularly, tumour lysis syndrome (TLS), a potentially life-threatening condition, has been reported in venetoclax-treated patients [60,61]. Kidney damage is a serious long-term effect of venetoclax-related TLS. Moreover, venetoclax induces cardiotoxicity through the modulation of oxidative-stress-mediated cardiac inflammation and apoptosis via the NF-kB and BCL-2 pathways [62]. Additional adverse reactions include gastrointestinal disorders (diarrhea, nausea, constipation, and vomiting) [63]. Notably, some side effects, such as renal dysfunction, hepatotoxicity, and cytopenias, may persist after patients stop venetoclax treatment [64]. To mitigate these adverse effects, venetoclax is dispensed in increasing weekly doses during treatment cycles (cycle 1: 20 mg/day (days 1–7), 50 mg/day (days 8–14), 100 mg/day (days 15–21), 200 mg/day (days 22–28); subsequent cycles are 400 mg/day (days 1–28)) [64]. Increasingly, studies are assessing strategies for the safety and efficacy of venetoclax administration. The findings in this study suggest that MP-A08 liposomes deliver MP-A08 at a significantly lower effective dose (80 times less) and venetoclax at lower doses (5 times less), identifying potential risk stratification for improved venetoclax efficacy and reduced toxicities that may enhance safety.

## 5. Conclusions

Nanocarriers have exhibited promising features in cancer drug administration, showcasing minimal toxicity and enhanced drug solubility, stability, and permeability. In a previous study, we demonstrated that liposomal encapsulation of MP-A08 improved its potency and efficacy in targeting SPHK1, leading to enhanced AML cell death [17]. In this study, we further demonstrated that the combination therapy of MP-A08 liposomes with cytarabine showed synergistic activity against AML cells in vitro. Similarly, the dual therapy of MP-A08 liposomes and venetoclax induced synergistic apoptotic cell death in vitro. These findings suggest that the nanoformulation of MP-A08 could serve as a foundation for more effective AML treatments, particularly in combination therapy to increase the efficacy of cytarabine or venetoclax, and, importantly, to reduce the adverse toxicities associated with these drugs. Overall, the fundamental foundational work in this study represents a significant step in addressing the challenges associated with the delivery of MP-A08 as an anti-cancer agent and highlights the potential of combination therapies to improve the safety and efficacy of AML treatment. Future studies should involve conducting in vivo assessments of these combination therapies using orthotopic AML models. For these studies, human AML cells will be engrafted into mice, generating a human AML cancer model. Subsequently, we will administer the combination treatments to mice, evaluating both survival and an elective cull analysis to measure human AML leukemic burden and therapeutic efficacy. These pre-clinical in vivo assessments will be crucial for validating the efficacy and safety of these combination strategies, aiming to provide therapeutic regimens to sensitize AML cells to these drugs, potentially overcoming existing resistance and allowing patients to achieve a deeper and more prolonged first remission.

## Data Availability

The data presented in this study are available in this article.
